# RACE-ETHNICITY AND HOPELESSNESS IN OLDER AMERICANS: WHO’S AT GREATEST RISK AND WHY?

**DOI:** 10.1093/geroni/igz038.1631

**Published:** 2019-11-08

**Authors:** Uchechi Mitchell

**Affiliations:** University of Illinois at Chicago, Chicago, Illinois, United States

## Abstract

Racial and ethnic minorities are more likely to experience adversity throughout their lives, which puts them at greater risk feelings of despair and powerlessness. This study uses data from 5,500 respondents from the Health and Retirement Study to assess racial/ethnic differences in hopelessness and test whether older blacks and Hispanics experience greater increases in hopelessness as they age. Hopelessness was assessed using 4-items that capture the extent to which a person has a negative outlook towards the future and believes they are powerlessness to overcome the obstacles they face; it is measured at three time points: 2006/2008, 2010/2012 and 2014/2016. Linear regression models were used to assess differences in hopelessness by race/ethnicity and linear mixed models were used to assess racial/ethnic differences in trajectories of hopelessness over time. Older blacks and Hispanics were more likely to report feelings of hopelessness at each timepoint of the study. Differences between blacks and whites were completely explained by differences in education and poverty status, while differences between Hispanics and whites remained. Although minority elders had higher levels of hopelessness at each time point, older whites experienced steeper increases in hopelessness over time. These findings suggest that structural factors influence feelings of hopelessness among minority elders. However, older blacks and Hispanics may develop resilience to hopelessness as they age.

